# Factors associated with meeting the recommendations for physical activity, sedentary behavior, and sleep in adolescents: a cross-sectional study

**DOI:** 10.1590/1984-0462/2026/44/2025027

**Published:** 2026-01-26

**Authors:** Karoline Barreto da Silva Rocha, Samanta Barbosa Feitosa, Rildo de Souza Wanderley, André dos Santos Costa, Valter Cordeiro Barbosa, Mauro Virgílio Gomes de Barros, Carla Menêses Hardman, Daniel da Rocha Queiroz

**Affiliations:** a Universidade Federal de Pernambuco, Recife, PE, Brazil.; b Universidade de Pernambuco, Recife, PE, Brazil.; c Universidade Estadual do Cearø, Fortaleza, CE, Brazil.

**Keywords:** Physical activity, Sedentary behavior, Sleep, Life style, Adolescent, Atividade física, Comportamento sedentário, Sono; Estilo de vida, Adolescente

## Abstract

**Objective:**

To analyze the association between the Social Ecological Model and meeting the physical activity, sedentary behavior, and sleep recommendations in a combined and integrated manner among adolescents.

**Methods:**

This is a cross-sectional study conducted in public schools in the Metropolitan Region of Recife, state of Pernambuco, Brazil, with adolescents aged 14 to 17 years. An adapted version of the Global School-based Student Health Survey was used as the instrument. Robust Poisson regressions were performed.

**Results:**

Approximately 1.8% of the 576 adolescents met all three recommendations simultaneously. Enjoying physical activity (prevalence ratio [PR] 11.62; 95% confidence interval [CI] 1.50–89.75) was associated with the combined adherence to the physical activity and sedentary behavior recommendations. Having two or more friends (PR 0.38; 95%CI 0.18–0.76) and participating in one (PR 0.39; 95%CI 0.20–0.78) or two physical education classes per week (PR 0.43; 95%CI 0.20–0.92) were associated with lower probabilities of non-compliance with these recommendations. Self-rated sleep quality as good (PR 2.49; 95%CI 1.09–5.67) was associated with higher prevalence of meeting the combined recommendations for sedentary behavior and sleep. Being male (PR 0.48; 95%CI 0.23–0.97) and participating in one physical education class per week (PR 0.40; 95%CI 0.16– 0.99) were associated with lower prevalence of not meeting the recommendations for sedentary behavior and sleep. Self-reported sleep quality as good (PR 3.11; 95%CI 1.36–7.10) as well as being male (PR 2.10; 95%CI 1.14–3.87) were associated with a higher likelihood of meeting the combined recommendations for physical activity and sleep. Actively commuting to school (PR 0.48; 95%CI 0.27–0.83) was associated with a lower likelihood of not meeting these recommendations.

**Conclusions:**

Intrapersonal, interpersonal, and community factors are associated with adherence to physical activity, sedentary behavior, and sleep recommendations in adolescents.

## RESUMO

**Objetivo:**

Analisar a associação entre o Modelo Social Ecológico e o atendimento das recomendações de atividade física, comportamento sedentário e sono, de forma combinada e integrada, entre adolescentes.

**Métodos:**

Trata-se de um estudo transversal realizado em escolas públicas da Região Metropolitana do Recife (PE), com adolescentes de 14 a 17 anos. Utilizou-se uma versão adaptada do Global Schoolbased Student Health Survey como instrumento. Foram realizadas regressões de Poisson robustas.

**Resultados:**

Aproximadamente 1,8% dos 576 adolescentes atenderam às três recomendações simultaneamente. Gostar de atividade física (razão de prevalência [RP] 11,62; intervalo de confiança [IC]95% 1,50–89,75) foi associado ao cumprimento combinado das recomendações de atividade física e comportamento sedentário. Ter dois ou mais amigos (RP 0,38; IC95% 0,18–0,76) e participar de uma (RP 0,39; IC95% 0,20–0,78) ou duas aulas de educação física por semana (RP 0,43; IC95% 0,20–0,92) foram associados a menores probabilidades de não cumprir essas recomendações. A autoavaliação da qualidade do sono boa (RP 2,49; IC95% 1,09–5,67) foi associada a maiores probabilidades de atender às recomendações de comportamento sedentário e sono de forma combinada. Ser do sexo masculino (RP 0,48; IC95% 0,23–0,97) e participar de uma aula de educação física por semana (RP 0,40; IC95% 0,16–0,99) foram associados a menores probabilidades de não cumprir as recomendações de comportamento sedentário e sono. A autoavaliação da qualidade do sono como boa (RP 3,11; IC95% 1,36–7,10), assim como ser do sexo masculino (RP 2,10; IC95% 1,14–3,87), estiveram associados a maiores probabilidades de atendimento combinado das recomendações de atividade física e sono. Deslocar-se ativamente para a escola (RP 0,48; IC95% 0,27–0,83) esteve associado à menor probabilidade de não cumprir essas recomendações.

**Conclusões:**

Fatores intrapessoais, interpessoais e comunitários estão associados ao cumprimento das recomendações de atividade física, comportamento sedentário e sono em adolescentes.

## INTRODUCTION

Scientific evidence indicates that, while physical activity (PA) is essential for health,^1^ it is also important to consider other components of the 24-hour day in an integrated manner, including sedentary behavior (SB) and sleep.^2^ These behaviors form a movement continuum,^3^ which contributes to the continuity and sustainability of an active and healthy lifestyle.^4^ Considering all these behaviors throughout the day reflects the premise that “the whole day matters” for health promotion, given the interdependent nature of these activities.^5^

In this context, it is suggested that these behaviors are interconnected, such that each has reciprocal effects on the others, supporting the idea that their associations with health should not be analyzed in isolation.^6^ When considering the set of 24-hour movement behaviors, the time dedicated to SB, sleep, and PA is strongly associated with positive health outcomes.^7^

Various guidelines provide recommendations on how these behaviors can be structured throughout the day and emphasize the importance of each behavior in promoting individual health.^8^ These include recommendations for a health promotion strategy in adolescents (14–17 years old), which include at least 60 minutes of moderate-to-vigorous PA (MVPA) per day, limiting screen-based SB to no more than 2 hours per day, and maintaining an adequate sleep routine of 8–10 hours per night.^8^

Adherence to the levels recommended in these guidelines is associated with better physical, mental, and social health indicators.^7^ However, adherence is alarmingly low in prevalence, particularly among adolescents in South America, where values range from 2.9 to 3.2%.^9^ Despite the significant health benefits of meeting recommendations for PA, SB, and sleep, evidence highlights that biological sex differences, environmental influences,^10^ and social and cultural factors^9^ may be associated with adherence to these recommendations. While studies have explored factors associated with one or two of these behaviors,^10^ few have investigated the factors influencing the integrated adherence (all three behaviors) to these recommendations during adolescence.^8^

Starting from this principle, the Social Ecological Model (SEM) adopts a comprehensive approach to understanding the factors that influence the adoption of healthy behaviors, such as PA, SB, and sleep, considering aspects ranging from individual factors to public policies.^11^ The choice of this model is based on its ability to integrate intrapersonal, interpersonal, organizational, community, and policy-level factors, allowing for a holistic analysis of the determinants associated with these behaviors. From this perspective, the application of the SEM requires a context that emphasizes the interaction among different levels of influence,^11^ as occurs in large urban centers. Thus, analyzing adolescents from the Metropolitan Region of Recife (state of Pernambuco, Brazil), characterized by high population density, marked social inequalities, and environmental diversity becomes particularly relevant, since these conditions may affect the amount of PA, SB, and sleep duration.

Despite the relevance of the topic, no studies to date have simultaneously addressed these behaviors among adolescents in the Metropolitan Region of Recife, nor investigations that consider the joint compliance with PA, SB, and sleep recommendations. Thus, the current study aimed to analyze whether there is an association between the SEM and adherence to recommendations for PA, SB, and sleep in a combined (paired behaviors) and integrated way among adolescents.

## METHOD

This study is a school-based cross-sectional epidemiological investigation conducted among students from high school classes in public schools in the Metropolitan Region of Recife. The research was performed following the criteria established by the Strengthening the Reporting of Observational Studies in Epidemiology (STROBE).^12^ This is a secondary analysis of a study entitled: “Association between physical activity, sedentary behavior, motor competence, sleep quality, and adiposity indicators in high school students from the Metropolitan Region of Recife”.

Adolescents aged 14 to 19 years, of both sexes, participated in the study. However, eligibility was restricted to adolescents aged 14 to 17 years, in accordance with the established recommendations.^8^ Additionally, participants who reported PA, SB, or sleep time exceeding 24 hours per day were excluded from the study. Thus, the mathematical expression “24h–TS>MVPA+ST” was established to determine whether adolescent data should be excluded. In this formula, sleep time (TS) was subtracted to examine if the remaining time, encompassing MVPA combined with screen time (ST), was greater than the total period spent awake. For the data to be considered valid, the total waking time (24 hours minus sleep time) must exceed the combined duration of MVPA and ST.

The sample selection occurred in two stages, with the first sampling unit being schools and the second being classes (at high school level). The Research Randomizer program was used to select the sampling units. Data collection was conducted in 20 randomly selected schools. This selection was based on the Regional Education Management Offices and considered four schools located in the North Metropolitan, four in the South Metropolitan, six in Recife North, and six in Recife South. The sample included one small school, 15 medium-sized schools, and four large schools.

The sample size was calculated using a 2% maximum margin of error and an expected frequency of 50%. For associations, it aimed to detect prevalence ratios ≥1.2 with 95% confidence interval and 80% statistical power. A design effect of 1.5 was applied. Using the OpenEpi software, considering the number of students enrolled in 2022 (n=113,210), the required sample size was estimated at 575 participants.

A self-administered version of the Global School-based Student Health Survey questionnaire was used as the data collection instrument. The instrument was administered via tablets, with an average application time of 40–50 minutes per participant and reproducibility coefficients ranging from 0.77–1.00. Tie questionnaire data were tabulated using SPHYNX^®^ software. All statistical analyses were performed using Stata, version 17.0.

The independent variables corresponding to the SEM were operationalized. The variables in the intrapersonal domain included; sex (0=Male; 1=Female), age (0=14–15 years; 1=16–17 years), skin color (0=White; 1=Non-white), liking physical activities (0=Like PA/Neither like nor dislike; 1=Dislike PA), and sleep quality (0=Poor/Regular; 1=Good/Very good/Excellent). Interpersonal variables were operationalized as follows: mother’s education level (0=≤8 years; 1=>8 years), participation in physical education classes (0=None; 1=1 time per week; 2=2, 3, or more), and number of close friends (0=None; 1=1; 2=2, 3, or more).

The organizational variables were operationalized as follows: evaluation of the school’s group of teachers and administrators (0=Good/Very good; 1=Poor/Regular) and provision of physical education classes (0=None; 1=1 time per week; 2=2, 3, or more). Additionally, the community variables included the location of residence (0=Urban; 1=Rural) and mode of transportation to school (0=Active transportation; 1=Passive transportation). This variable was dichotomized, with active commuting referring to travel on foot or by bicycle, and passive commuting referring to traveled by car, motorcycle, or bus. Finally, in the group of variables related to public policies, the school shift was included (0=Semi-integral; 1=Morning, Afternoon, or Evening; 2=Integral).

The dependent variables of the study were operationalized according to the recommendations for PA, SB, and sleep.^8^ PA was assessed based on self-reported MVPA in days and minutes during a typical week and the preceding week. Both were converted to total weekly minutes, and the average of the two values was used as the final measure. Adolescents were classified as “meeting recommendations” (≥420 min/week).^8^ SB was estimated by accumulating recreational ST (TV, smartphone/tablet, computer, or video games). This variable was measured based on weekdays and weekends and reported in daily hours and minutes. The adopted cut-off points were: ≤2 hours/day (meeting the recommendation). Sleep was assessed based on the number of hours reported for weekdays and weekends. For this variable, meeting the recommendations corresponded to 8–10 hours/day.

Statistical power was calculated using OpenEpi for a cross-sectional study with 576 individuals and a 95% confidence interval. The prevalences entered into OpenEpi were pairs of compliance with recommendations, considering the respective values for exposed (E) and non-exposed (NE) individuals. Thus, PA+SB (E: 8.5%), SB+sleep (E: 6.0%), PA+sleep (E: 8.0%) and two or more (E: 19.1%) were considered for this calculation. The estimated statistical power was 100%, indicating adequacy to detect associations.

Categorical variables were presented as absolute and relative values with their respective confidence intervals. To explore how the different SEM strata influence adherence to the recommendations, robust Poisson regressions were applied. Data analysis was conducted hierarchically, incorporating all the SEM strata in successive stages. The “enter” method was applied at each stage, simultaneously including all independent variables within each stratum. Model fit quality was assessed using Deviance and Pearson χ^2^ tests. The magnitude of association was estimated using prevalence ratios and 95% confidence intervals. All analyses were adjusted for all SEM strata, adopting a significance level of p<0.05.

The current study was approved by the Research Ethics Committee at the Federal University of Pernambuco under opinion nº 5.921.335. Furthermore, the study followed the guidelines of the National Health Council for research with human beings, ensuring the ethics and integrity of the research. Participation in the research was conditioned on the return of the free and informed consent form and the free and informed assent form, signed.

## RESULTS

**Figure 1.**
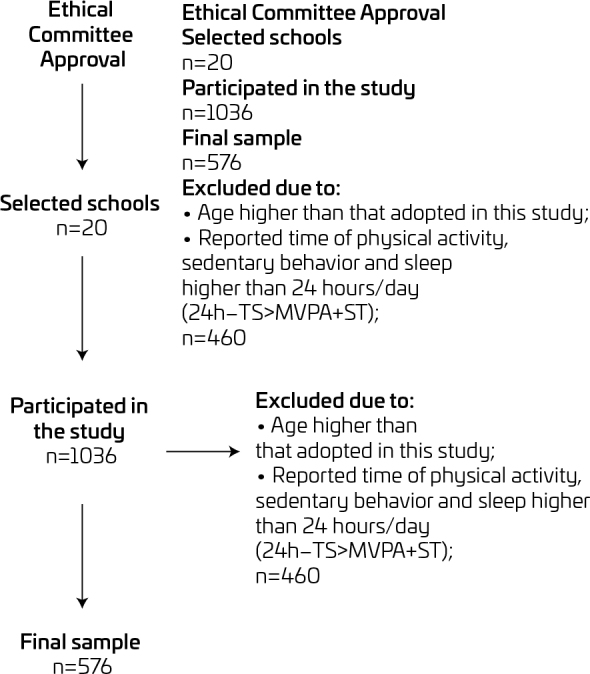
Flowchart of participant selection.

TS: sleep time; MVPA: moderate-to-vigorous physical activity; ST: screen time.

A total of 1036 students participated in the study; however, after applying the eligibility criteria, the final sample consisted of 576 adolescents (Table 1). To meet the pre-defined criteria for the sample, 460 participants were excluded (Figure 1).

**Table 1.** Sociodemographic characteristics of high school students from the State Network in the Metropolitan Region of Recife (PE), Brazil.

Variables	Total (n=576)		Female (n=315)		Male (n=261)	
	n	%	n	%	n	%
Age group (years)						
14–15	121	21.1	74	23.5	47	18.0
16–17	455	78.9	241	76.5	214	82.0
Skin color						
White	137	23.8	83	26.4	54	20.6
Non-white	439	76.2	232	73.6	207	79.4
Like physical activity						
Yes	127	22.0	99	31.5	28	10.7
No	449	78.0	216	68.5	233	89.3
Sleep quality						
Good	345	59.9	163	51.8	182	69.7
Bad	231	40.1	152	48.2	79	30.3
Mother’s education (years)						
≤8	239	41.4	135	42.8	104	39.8
>8	337	58.6	180	57.2	157	60.2
Participation in PE classes (week)						
0	128	22.2	90	28.5	38	14.5
1	234	40.7	130	41.2	104	40.0
2 or more	214	37.1	95	30.3	119	45.5
Number of friends						
0	23	4.0	12	3.8	11	4.2
1	66	11.4	38	12.0	28	10.8
2 or more	487	84.6	265	84.2	222	85.0
Evaluation of pedagogical team						
Good	371	64.4	198	62.8	173	66.2
Bad	205	35.6	117	37.2	88	33.8
Offer of PE classes (week)						
0	60	10.4	34	10.9	26	9.9
1	339	58.9	188	59.6	151	58.0
2 or more	177	30.7	93	29.5	84	32.1
Residence location						
Urban	502	87.1	277	87.9	225	86.2
Rural	74	12.9	38	12.1	36	13.8
Active transportation						
Yes	163	28.3	96	30.5	67	26.0
No	412	71.7	219	69.5	193	74.0
Schooling shift						
Semi/full-time	203	35.2	115	36.5	88	33.8
Morning, afternoon, or evening	81	14.0	44	14.0	37	14.1
Full-time	292	50.8	156	49.5	136	52.1

PE: physical education.

Descriptive statistics are presented in Table 1. Table 2 shows the mean and standard deviation for PA, weekday ST, weekend ST, weekday sleep, and weekend sleep. Descriptive statistics are also presented for isolated behaviors, combined behaviors, and integrated behavior. Table 3 presents the associations between the SEM strata and recommendations for PA, SB, and sleep in adolescents, in both an integrated and combined forms.

**Table 2.** Isolated, combined, and integrated care of physical activity, sedentary behavior, and sleep recommendations in high school adolescents from the State Network of the Metropolitan Region of Recife (PE), Brazil.

Variables	Total (n=576)		Female (n=315)		Male (n=261)	
	Mean	SD	Mean	SD	Mean	SD
Physical activity (min)	36.4	32.8	28.2	29.7	46.3	33.6
Weekday screen time (min)	165.9	111.7	171.4	114.6	159.4	107.9
Weekend screen time (min)	567.7	370.5	587.4	359.4	544.0	382.9
Weekday sleep (min)	410.6	86.6	404.5	89.3	417.9	82.9
Weekend sleep (min)	483.8	108.6	481.5	113.9	486.6	102.1
	**n**	**%**	**n**	**%**	**n**	**%**
Physical activity						
Yes	171	30	56	18	115	44
No	405	70	259	82	146	56
Sedentary behavior						
Yes	137	24	69	22	68	25
No	439	76	246	78	193	75
Sleep						
Yes	157	27	89	28	68	26
No	419	73	226	72	193	74
PA+SB						
Yes	49	8.5	20	6.4	29	11
No	527	91.5	295	93.6	232	89
SB+sleep						
Yes	35	6	23	7.3	12	4.6
No	541	94	292	92.7	249	95.4
PA+sleep						
Yes	46	8	15	4.7	31	12
No	530	92	300	95.3	230	88
Meets 2 or more						
Yes	110	19.1	46	14.6	64	24.5
No	466	80.9	269	85.4	197	75.5
Meets 3						
Yes	10	1.8	6	2	4	1.5
No	566	98.2	309	98	257	98.5

SD: standard deviation; min: minutes; PA: physical activity; SB: sedentary behavior.

**Table 3.** Association between the factor groups of the Social Ecological Model and compliance with the combined and integrated recommendations for physical activity, sedentary behavior, and sleep among adolescents in the Metropolitan Region of Recife.

Variables	PA +SB			SB+SLEEP			PA+SLEEP			TWO OR MORE		
	PR (95%CI)	SE	p-value	PR (95%CI)	SE	p-value	PR (95%CI)	SE	p-value	PR (95%CI)	SE [	p-value
Sex												
Male	1.28 (0.74–2.21)	0.35	0.37	**0.48 (0.23–0.97)**	**0.17**	**0.04**	**2.10(1.14–3.87)**	**0.65**	**0.01**	1.31 (0.91–1.87)	0.23	0.13
Age group (years)												
16–17	2.02 (0.84–4.81)	0.89	0.11	1.02 (0.46–2.26)	0.41	0.95	1.47 (0.68–3.19)	0.58	0.31	1.50 (0.91–2.48)	0.38	0.10
Race												
Non-white	1.55 (0.75–3.19)	0.57	0.22	0.64 (0.31–1.32)	0.23	0.23	1.26 (0.62–2.53)	0.44	0.51	0.89 (0.60–1.31)	0.17	0.56
Like physical activity												
Yes	11.6 (1.50–89.7)	12.12	**0.01**	2.23 (0.79–6.29)	1.18	0.12	1.46 (0.57–3.71)	0.69	0.42	**2.93 (1.44–5.98)**	**1.06**	**<0.01**
Sleep quality												
Good	0.74 (0.43–1.27)	0.20	0.28	**2.49 (1.09–5.67)**	**1.04**	**0.03**	**3.11(1.36–7.10)**	**1.31**	**<0.01**	1.47 (0.99–2.19)	0.29	0.05
Mother’s education level (years)												
>8	1.31 (0.75–2.30)	0.97	0.33	1.24 (0.60–2.54)	0.45	0.55	1.69 (0.91–3.13)	0.53	0.09	1.29 (0.90–1.84)	0.23	0.15
Participation in PE (week, class)												
1 class	1.74 (0.72–4.22)	0.78	0.21	2.62 (0.97–702)	1.31	0.05	0.70 (0.30–0.48)	0.30	0.41	1.23 (0.72–2.12)	0.34	0.44
2 or more	1.79 (0.75–4.27)	0.79	0.18	1.89 (0.66–5.45)	1.02	0.23	1.08 (0.48–2.43)	0.44	0.84	1.31 (0.76–2.27)	0.36	0.32
Number of friends												
1	0.38 (0.12–1.18)	0.22	0.09	0.80 (0.17–3.65)	0.62	0.77	1.06 (0.25–4.49)	0.78	0.92	0.61(0.28–1.34)	0.24	0.22
2 or more	**0.38(0.18–0.76)**	**0.13**	**<0.01**	0.67 (0.17–2.60)	0.46	0.56	0.81 (0.23–2.79)	0.51	0.74	0.61 (0.33–1.12)	0.19	0.11
Evaluation of pedagogical team												
Good	1.58 (0.88–2.86)	0.47	0.12	0.71 (0.36–1.37)	0.23	0.31	1.18 (0.64–2.17)	0.36	0.57	1.19 (0.82–1.74)	0.22	0.34
Offer of PE (week, class)												
1	0.39 (0.20–0.78)	0.13	<0.01	0.40 (0.16–0.99)	0.18	0.04	0.82 (0.36–1.89)	0.34	0.65	0.71 (0.42–1.21)	0.19	0.21
2 or more	**0.43 (0.20–0.92)**	**0.16**	**0.03**	0.40 (0.14–1.14)	0.21	0.08	0.55 (0.21–1.38)	0.25	0.20	0.76 (0.42–1.36)	0.22	0.36
Residence location												
Rural	1.33 (0.61–2.88)	0.52	0.46	0.98 (0.36–2.64)	0.49	0.96	0.89 (0.37–2.12)	0.39	0.80	0.84 (0.52–1.38)	0.21	0.51
Active transportation												
Yes	1.02 (0.58–1.77)	0.28	0.93	0.94 (0.47–1.89)	0.33	0.87	**0.48 (0.27–0.83)**	**0.13**	**0.01**	0.85 (0.59–1.23)	0.15	0.40
Schooling shift												
Morning, afternoon, or evening	1.58 (0.65–3.83)	0.71	0.30	1.05 (0.39–2.79)	0.52	0.91	0.72 (0.29–1.75)	0.32	0.47	0.88 (0.53–1.48)	0.23	0.65
Full-time	1.52 (0.63–3.63)	0.67	0.34	0.88 (0.35–2.20)	0.41	0.78	1.15 (0.50–2.62)	0.48	0.73	0.91 (0.55–1.49)	0.22	0.71

PA: physical activity; SB: sedentary behavior; PR: prevalence ratio; SE: standard error; PE: physical education; df: degrees of freedom.

Deviance and Pearson χ^2^ test for model adjustment: PA+SB (Deviance/df=1.0; Pearson χ^2^/df=0.40); SB+SLEEP (Deviance/df=1.0; Pearson χ^2^/df=0,97); PA+SLEEP (Deviance/df=1.0; Pearson χ^2^/df=0.71); Two or more (Deviance/df=1.0; Pearson χ^2^/df=0,99). The values highlighted in bold indicate a statistically significant difference.

Enjoying PA was associated with compliance with the combined recommendations for PA and SB (PR 11.62; 95%CI 1.50– 89.75). Having two or more close friends reduced the likelihood of not meeting PA and SB recommendations by 62% (PR 0.38; 95%CI 0.18–0.76). Participating in at least one physical education class per week was associated with a lower probability of not meeting these recommendations (PR 0.39; 95%CI 0.20–0.78). Furthermore, participating in two or more classes per week also reduced this probability (PR 0.43; 95%CI 0.20–0.92).

Self-rated good sleep quality was associated with a higher probability of meeting the combined recommendations for SB and sleep (PR 2.49; 95%CI 1.09–5.67). Additionally, being male was associated with a lower probability of meeting these recommendations (PR 0.48; 95%CI 0.23–0.97). Furthermore, participating in at least one physical education class per week (PR 0.40; 95%CI 0.16–0.99) was associated with a lower probability of not meeting the combined recommendations for SB and sleep.

Finally, self-rated good sleep quality was associated with higher compliance with the combined recommendations for PA and sleep (PR 3.11; 95%CI 1.36–7.10). Moreover, active commuting to school (PR 0.48; 95%CI 0.27–0.83) was associated with a lower probability of not meeting these recommendations, whereas being male (PR 2.10; 95%CI 1.14–3.87) was associated with a higher probability of meeting the combined recommendations.

## DISCUSSION

This study aimed to analyze the association between the SEM and adherence to PA, SB, and sleep recommendations in an integrated and combined manner among adolescents. The findings showed that 1.8% of adolescents adhered to all three recommendations, similar to a systematic review^9^ that reported 2.6% adherence among adolescents from 23 countries. While full adherence was low and not significant, adherence to two or more recommendations was statistically significant, reflecting common combinations of behaviors, often including at least one unhealthy behavior.^10^

Adolescents who reported enjoying PA showed higher adherence to the combined PA and SB recommendations, corroborating findings from a Canadian study.^13^ Combined adherence to these recommendations was linked to younger age, male sex, white ethnicity, higher socioeconomic status, and a lower prevalence of overweight/obesity.^13^

At least one weekly physical education class in schools showed a protective effect on combined PA and SB, suggesting a lower prevalence of non-adherence. Literature indicates similar associations with MVPA and reduced SB, highlighting schools and physical education classes as important strategies and supporting public policies to promote regular adolescent PA.^14,15^

Having more friends showed a protective effect, as physically active peers positively influence behaviors, encouraging PA, reducing sedentary time, and supporting adequate sleep, emphasizing the importance of peer support in promoting healthy movement behaviors.^16,17^ Adolescents who reported good sleep quality were more likely to meet combined SB and sleep recommendations. Excessive ST is associated with poorer sleep quality due to the negative impact of blue light on sleep cycles.^18^ Conversely, adolescents who meet sleep recommendations tend to report positive perceptions of this behavior, which is also linked to reduced ST and prolonged SB.^19^

Being male was associated with adherence to combined SB and sleep. This finding may be explained by behavioral differences between sexes, as boys tend to engage more in PA and are less exposed to prolonged SB compared to girls.^20^ Social norms often encourage girls to adopt less physically active behaviors, while boys typically participate in more dynamic activities.^10^

Positive self-assessment of sleep quality was associated with meeting combined PA and sleep. This may be justified by the interaction between sleep and PA, where individuals who wake up earlier are more likely to engage in PA, contributing to adherence to recommendations.^21^ Furthermore, the association between PA and adequate sleep duration may be related to increased energy expenditure from PA, necessitating appropriate recovery through sleep.^18^

Active commuting to school was associated with adherence to PA and sleep, as it increases daily PA and helps establish a structured routine.^22^ This promotes regular sleep and wake times, supporting sleep recommendations and facilitating both an active lifestyle and improved sleep patterns in adolescents.^23^

The association between being male and adherence to combined PA and sleep may reflect higher energy expenditure in boys, which increases the need for recovery.^18^ Additionally, social norms encourage boys’ participation in PA, while girls often engage more in SB, which may impair sleep and hinder adherence to combined recommendations.^10^

Several social and cultural factors influence behavioral patterns related to PA and SB, encompassing levels of the SEM.^24^ A systematic review identified some barriers: in the interpersonal domain, the absence of support from peers, family members, and teachers, as well as living with individuals who do not follow healthy routines, negative experiences, and financial limitations, constitute significant barriers.^24^ Environmental factors include concerns about safety in outdoor areas, limited access to sports programs, school curricula focused on sports perceived as masculine, and the transition from childhood to adolescence.^25,26^

In the present study, boys were more likely to meet the combined PA and sleep requirements, but less likely to meet the recommendations for low SB and sleep. Systematic review evidence shows that boys often exhibit high PA, while girls tend to have longer sleep duration.^10^ These differences likely stem from both biological and sociocultural factors. Social norms may encourage boys to be more active, whereas girls often engage in more sedentary or differently structured activities.^10^ Sex-influenced behavioral tendencies emphasize the need for health promotion strategies tailored to males and females. Interventions should address unique obstacles and social environments to encourage healthier 24-hour movement behaviors.^10,26^ Generic guidelines may be insufficient; approaches recognizing socially influenced behaviors are essential for meaningful public health improvements in this population.^26^

Adolescent males showed lower compliance with combined recommendations for low SB and sleep, partly due to high ST and associated behavioral clusters. Social and cultural norms may also encourage extensive screen use among boys.^10^ Sex-sensitive strategies should focus on reducing SB, increasing PA, and improving sleep, particularly among boys. Given this context, health promotion strategies should address sex inequalities by creating safe, equitable school environments, inclusive curricula, and involving qualified professionals to encourage girls’ participation in sports and PA.^27^

Adolescents who reported enjoying PA showed higher adherence to two or more recommendations, corroborating previous findings.^28^ The intrinsic pleasure and motivation to engage in PA favor the adoption of healthy behaviors, as individuals with a positive self-perception of health and motivation are more likely to adhere to all three recommendations in an integrated manner.^29^ Despite advances in research on 24-hour movement behaviors, most studies have focused on high-income populations. Understanding these factors in low- and middle-income countries is key to preventing low PA, high SB, and poor sleep.^30^

The study’s limitations include its cross-sectional design, which prevents the establishment of cause-and-effect relationships, and the use of self-reported measures for key variables. Recall bias may affect reports of PA, SB, and sleep, potentially compromising data accuracy. The results of the present study, although valid for the analyzed sample, may not be generalizable to all adolescents in the Metropolitan Region of Recife. To strengthen the findings, it is suggested that future studies address different socioeconomic profiles to obtain a more comprehensive understanding. Despite its limitations, this study is notable for analyzing combined factors related to PA, SB, and sleep among adolescents in a developing country. This multifactorial approach enhances understanding of related factors and supports more effective health promotion strategies using a valid and reproducibility instrument for epidemiological studies.

Future research should adopt a longitudinal design to investigate these associations, prioritizing populations in developing countries. Furthermore, the use of objective measures is recommended, as well as the inclusion of variables that proportionally represent all SEM levels, in order to ensure a more comprehensive and robust analysis. Moreover, compositional data analysis techniques are suggested, as they account for the 24-hour allocation of time across different movement behaviors.

In conclusion, intrapersonal, interpersonal, and community factors were significantly associated with compliance with the combined recommendations for PA, SB, and sleep. These findings emphasize the importance of individual aspects in promoting healthy habits, suggesting that both personal and environmental characteristics play a crucial role in adopting health-promoting behaviors in this population.

## Data Availability

The database that originated the article is available with the corresponding author.
